# Analysis of Opioid-Related Adverse Events in Japan Using FAERS Database

**DOI:** 10.3390/ph16111541

**Published:** 2023-11-01

**Authors:** Risako Hirai, Yoshihiro Uesawa

**Affiliations:** Department of Medical Molecular Informatics, Meiji Pharmaceutical University, Tokyo 204-8588, Japan

**Keywords:** opioids, adverse effect database, FDA Adverse Event Reporting System, reporting odds ratio, cluster analysis

## Abstract

Adverse events associated with opioid use in palliative care have been extensively studied. However, predicting the occurrence of adverse events based on the specific opioid used remains difficult. This study aimed to comprehensively analyze the adverse events related to µ-opioid receptor stimulation of opioids approved in Japan and investigate the tendencies of adverse event occurrence among different opioids. We utilized the FDA Adverse Event Reporting System database to extract reported adverse events for opioids approved in Japan. Cluster analysis was performed on reporting odds ratios (RORs) of adverse event names among opioids to visualize relationships between opioids and adverse events, facilitating a comparative study of their classifications. We calculated the RORs of adverse events for the target opioids. Cluster analysis based on these RORs resulted in five broad clusters based on the reported adverse events: i.e., strong opioids, weak opioids, loperamide, tapentadol, and remifentanil. This study provides a comprehensive classification of the association between μ-opioid-receptor-stimulating opioids and adverse events.

## 1. Introduction

Opioids are important agents in the field of palliative medical treatment because of their powerful analgesic properties [1]. In the management of pain in particular, including cancer pain, opioids are key to improving patient quality of life. Although opioids are an important class of drugs in palliative care worldwide, opioid sales in the United States increased fourfold between 1999 and 2010 [2], causing a social problem known as the opioid crisis. In response, the World Health Organization (WHO) published the guidelines “Ensuring Balance in National Policies on Controlled Substances” in 2011 and “WH Guidelines on the Availability and Accessibility of Controlled Substances” in 2012 [3]. Regulation of controlled substance prescriptions has consequently been thoroughly enforced in the USA, and the prescribing and use of opioids have declined [4]. However, the number of opioid-related overdose deaths increased from 2010 to 2017 and stabilized until 2019. Then, a significant increase was observed in 2020 (68,630 deaths) and again in 2021 (80,411 deaths) [4].

In contrast, Japan has yet to experience such an opioid crisis. On the contrary, the current situation is one in which the quantity of opioids prescribed is not commensurate with patients’ pain due to doctors’ reluctance to prescribe opioids. This situation has been called into question by those who point out that opioid use in Japan tends to be low, both in general and in comparison to use in other countries [5].

Japan lags behind the USA not only in the prescription and use of opioids but also in the approval of opioid drugs. For example, tapentadol was approved for prescription in the USA in November 2008 [6] but was approved in Japan only in August 2014 [7]. Hydromorphone was approved in the USA in 1984 [8] but remained unapproved in Japan for over 30 years. Finally, after forceful requests from the Japanese Society for Palliative Medicine and others, the brand name Nalrapide^®^/Hydromorphone Hydrochloride was approved in Japan in June 2017 [9]. Because the approval of opioids in Japan is slower than in the USA, studies on adverse events tend to accumulate fewer cases in Japan than in the USA.

For these reasons, it is difficult to secure sufficient cases for robust analysis either in opioid case studies or using the Japanese Adverse Drug Event Report database (JADER), a large database in Japan. Although opioid prescription availability and guidelines vary from country to country as described above, Japanese guidelines for palliative medicine specify clearly how to respond to opioid side effects [10]. In clinical practice, coping therapies are used to deal with various symptoms of opioid side effects [10].

Known adverse events to opioids from a pharmacological point of view include nausea and vomiting, which are believed to be caused by chemoreceptor trigger zone stimulation due to μ-opioid receptor stimulation [11], and constipation, which is believed to be influenced by the peristaltic inhibitory effect unique to opioids. Respiratory depression, drowsiness, delirium, and somnolence have been observed due to the inhibitory effect on the medullary respiratory control center [12,13]. Although the involvement of μ-opioid receptors has been pointed out in these side effects, the details of the mechanism of occurrence of each of these symptoms have not been elucidated [14,15,16,17,18,19,20,21]. It has been reported that differences in the propensity to develop adverse events exist among opioids relative to adverse events for which the occurrence mechanism is not yet fully understood [22]. Thus, the goal of this study is to classify the association between adverse events and opioids comprehensively by extracting and clustering the μ-opioid receptor stimulating opioids from the database. Therefore, for this study, we conducted an adverse event analysis of opioids using the FDA Adverse Event Reporting System (FAERS) [23,24,25,26,27,28,29,30], a large database in the United States because of the problem of opioid use in Japan, the relatively new drug, and the low number of cases.

We conducted a comprehensive analysis of USA-based adverse events due to μ-opioid receptor stimulation by opioids approved in Japan in order to compare the incidence of adverse events among opioids. We hope that our findings will assist in drug selection, opioid switching, and the appropriate use of opioids.

## 2. Results

### 2.1. Number of Reported Adverse Events

According to the data from FAERS, the opioids oxycodone, morphine, and fentanyl exhibited the highest number of reported adverse events. It is worth noting that these drugs are commonly used in palliative care settings [31,32]. Please refer to Table 1 and Table S1 for more information.

### 2.2. Top of the Adverse Event

We identified 47 preferred terms for adverse events based on the mean lnROR of the target opioids. Frequently observed side effects, such as somnolence, delirium, and constipation, which are known to be associated with µ-opioid receptor stimulation, were common among these adverse events. We extracted the top 47 adverse events that had been reported in >150,000 cases (Table 2). The ROR and 95% confidence intervals for each target opioid concerning the 47 adverse event terms with a positive average ROR are presented in the supplementary data. The table presents the top 47 adverse events with >150,000 reported cases, including columns for the adverse event, number of reports, average lnROR, and ROR. The ROR and 95% confidence interval for each target opioid concerning the 47 adverse event terms with a positive average ROR are presented in the supplementary data.

### 2.3. Hierarchical Cluster Classifications

**Figure 1:**
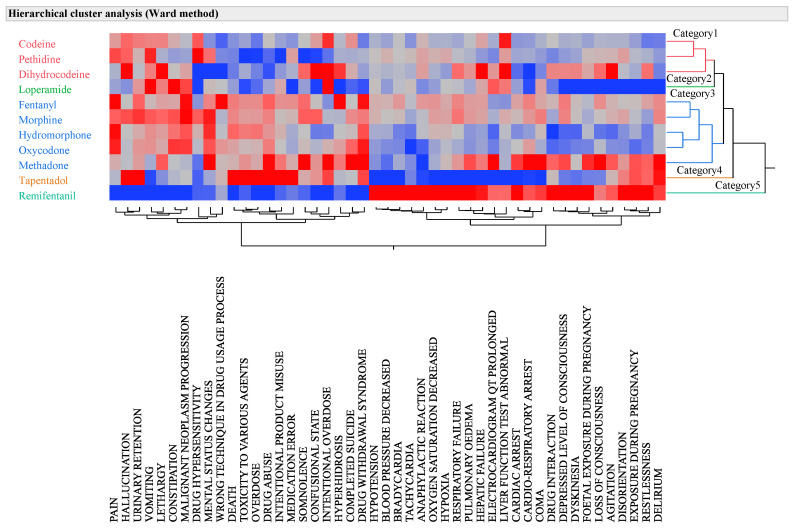
Cluster analysis based on lnROR of reported adverse event names for target opioids.

In this study, the 11 analyzed opioids were classified into five distinct groups using hierarchical cluster analysis (Ward’s method) based on the adverse event names and lnROR (Figure 1). The clusters comprised two primary groups of opioids, including loperamide, tapentadol, and remifentanil. Cluster 1 included codeine, pethidine, and dihydrocodeine. In cluster 2, loperamide was identified. Cluster 3 included fentanyl, morphine, hydromorphone, oxycodone, and methadone. Cluster 4 contained tapentadol, and cluster 5 contained remifentanil. Meanwhile, adverse effects were clustered into seven types. Cluster 1 included pain, malignant neoplasm progression, urinary retention, vomiting, constipation, hallucinations, altered mental status, lethargy, and the wrong technique in the drug usage process. Cluster 2 comprised death, drug toxicity, overdose, drug abuse, intentional misuse, and medical malpractice. Cluster 3 comprised somnolence, confusion, intentional overdose, hyperhidrosis, suicide, and drug withdrawal symptoms. Cluster 4 contained hypotension, bradycardia, tachycardia, an anaphylactic reaction, desaturation of oxygen, and hypoxia. Cluster 5 featured liver failure and abnormal liver function tests. Cluster 6 included echocardiographic (ECG) QT prolongation, cardiac arrest, and cardio-respiratory arrest. Cluster 7 encompasses disorientation, restlessness, and delirium.

The vertical axis represents the 11 µ-opioid receptor agonists, and the horizontal axis represents the names of reported adverse events. Red indicates a high log odds ratio and a high incidence of adverse events, whereas blue indicates a low log odds ratio and a low incidence of relevant adverse events.

We extracted primary and secondary suspect drugs from the drug table, removing duplicates. We then combined the reaction, demographic, and indication tables to create an analysis table.

The study utilized a 2 × 2 contingency table to investigate the association between reported adverse events and target opioids. The RORs were computed to determine the magnitude of this association.

## 3. Discussion

### 3.1. FAERS Database

This study analyzed the adverse event reports for target opioids using the FAERS database, which collects reports from public institutions in the United States. Although the focus was on opioids used in Japan, a larger number of cases were reported in FAERS compared with the JADER database, which is constructed by the Pharmaceuticals and Medical Devices Agency. This could be due to the reluctance of Japanese medical professionals to use opioids [33]. To enhance comparative validation among opioid drugs, the US side effect database was used.

As FAERS primarily reports adverse reactions from both inside and outside the United States, there are many cases of hydromorphone and tapentadol usage for which there is still limited information available in Japan. Consequently, FAERS analysis may contribute to palliative care in Japan. JADER, which is based on Japanese data, was not used in the analysis conducted in this study because the amount of data is less than that in FAERS; however, a study specific to the Japanese population would provide new knowledge and could guide treatment for the Japanese ethnic group. Thus, further data analysis and development of corresponding clinical research are desirable. Of the adverse events extracted using the ROR, those with a positive lnROR value were highly related to the target opioid. Adverse events such as somnolence, delirium, and constipation [10,11,12] were frequently reported in 47 diseases that were related to opioids (Table 2). We believe that the ROR of FAERS has a relationship with the side effects owing to the stimulation of the μ-opioid receptors. While it is generally not recommended to interpret ROR quantitatively in the analysis of side effect databases, we assumed that a highly reliable ROR that can withstand quantitative verification can be extracted using the number of reported adverse events and the *p*-value in Fischer’s exact test.

### 3.2. Categories of Opioids Based on Cluster Analysis

Cluster 1 included codeine, pethidine, and dihydrocodeine. This group tends to be less associated with adverse events such as overdose, substance abuse, and drug withdrawal symptoms compared with other groups. The reason is that drugs other than pethidine are clinically used for purposes other than narcotism and pain relief. Pethidine is used for purposes such as preanesthetic administration, adjunctive general anesthesia, and pain relief [34]. Adverse events such as overdose and substance abuse are classified into relatively small categories because they are often used under close supervision for purposes related to anesthesia.

Cluster 2 comprised loperamide, an over-the-counter drug commonly used to treat diarrhea symptoms. It is speculated that loperamide is closely related to diarrhea adverse events owing to its stimulation of peripheral μ-opioid receptors. Moreover, loperamide has a lower μ-opioid-receptor-stimulating effect in the central nervous system compared with other opioids, which suggests that it is less associated with adverse central nervous system events such as loss of consciousness and disorientation [35].

Cluster 3 comprised fentanyl, morphine, hydromorphone, oxycodone, and methadone, all classified as strong opioids used to manage cancer pain and chronic pain. These opioids have many reports of adverse events related to pain, and their association with malignant neoplasm progression and drug withdrawal syndrome was strongly classified. Methadone, also included in the same group, was associated with substance abuse, overdose, and drug withdrawal syndrome. This background is thought to be related to its use for heroin addiction treatment in the United States [36]. Methadone replacement therapy involves gradually withdrawing from the symptoms of addiction by administering methadone as a substitute for heroin.

Cluster 4 included tapentadol, which is expected to expand its efficacy range to include nervous system pain owing to its dual-acting mechanism that involves μ-opioid receptor agonistic action and noradrenaline reuptake inhibitory action [37]. Hence, it was clustered into a group distinct from other strong opioids. Tapentadol tends to be highly associated with overdoses and drug abuse [38], and its prescription for moderate or severe pain is approved, suggesting that it contributes significantly to improving the quality of life. The trend of other reported adverse events alludes to the fact that it is necessary to pay attention to delirium when using it, but it is possible that it is an opioid that can be used with relatively little effect on respiratory failure.

Cluster 5 contained remifentanil, which tends to cause fewer gastrointestinal adverse events, such as vomiting and constipation. A substantial tendency to develop respiratory failure, hypotension, and other adverse events was confirmed, which was different from that of other opioids. Remifentanil is used in the field of anesthesia and is dose- and rate-controlled for the induction and maintenance of anesthesia. This characteristic of remifentanil might have led to cluster classifications that differ from those of other opioids used for pain management [39].

### 3.3. Categories of Adverse Effects Based on Cluster Analysis

The adverse effects were clustered into seven types, each with specific characteristics and associated opioids:

- Cluster 1: Pain, progression of malignant neoplasms, urinary retention, vomiting, constipation, hallucinations, altered mental status, lethargy, errors in the drug use process. The above adverse events tended to be reported more frequently with morphine and less frequently with remifentanil. Frequent occurrences of vomiting and constipation are known as adverse events attributed to μ-opioid receptor stimulation of opioids [10]. Based on these results, morphine increases the risk of adverse effects due to decreased renal function, which leads to decreased elimination of the metabolite M6G [40]. This finding strongly confirms the strong association of morphine with vomiting and constipation, which was observed in many patients with deteriorated renal function.
- Cluster 2: Death, various drug toxicities, overdose, drug abuse, intentional misuse, medical malpractice. Among opioids, tapentadol was reported more frequently and remifentanil less frequently in this cluster, and tapentadol tended to be more involved in these adverse events than other strong opioids used in palliative care. The stronger association of tapentadol with drug abuse and overdose suggests that while tapentadol shows great promise for ease of use and efficacy in pain management, it should be used with caution due to its enhanced risks of illicit use.
- Cluster 3: Somnolence, confusion, intentional overdose, hyperhidrosis, suicide, and drug withdrawal symptoms. The above adverse events were found to cluster with a high incidence of methadone. An analytical study using the Australian database found that fentanyl and methadone were more frequently involved in unintentional intoxication than other opioids [41]. Our results seem to support the above studies.
- Cluster 4: Hypotension, bradycardia, tachycardia, anaphylactic reactions, decreased oxygen saturation, hypoxia. We observed a trend indicating a higher incidence for remifentanil and a lower incidence for tapentadol. As noted above, remifentanil is highly associated with intraoperative hypotension and the elevation of blood pressure, hence its use in anesthesiology. Remifentanil is an opioid used in an environment where it is prone to producing fluctuating circulatory dynamics that affect the supply–demand balance of oxygen supplied to the myocardium [42].
- Cluster 5: Liver failure, abnormal liver function tests. These adverse events comprised a cluster with a high incidence of dihydrocodeine and codeine. Codeine and dihydrocodeine tended to have higher incidence of reported adverse events, including abnormal liver function tests and liver failure. Currently, the association of opioid receptors with drug-induced liver injury is limited to the finding that opioid receptors are not present in the liver [43]. However, the involvement of diverse stress response pathways (e.g., pathways related to oxidative stress, inflammatory stress, DNA damage, folded proteins, heat shock, and apoptosis) dependent on drug species has been reported with respect to the development of liver injury [44]. Unfortunately, our knowledge of opioid-induced liver injury is limited. The cluster classification performed in this study demonstrated a significant association with liver injury relative to codeine and dihydrocodeine; thus, future findings are expected to be accumulated.
- Cluster 6: ECG QT prolongation, cardiac arrest, cardiac arrest—respiratory arrest. This cluster had a high incidence of methadone, and methadone tended to have a higher incidence of ECG QT prolongation, cardiac arrest, and cardiac arrest–respiratory arrest compared to other μ-opioid-receptor-stimulating opioids. The cluster was high in methadone, and methadone tended to have a higher incidence of ECG QT prolongation, cardiac arrest, and cardiac arrest–respiratory arrest compared to other μ-opioid-receptor-stimulating opioids. In addition, a previous study of methadone reported a stronger association with the adverse event of ECG QT prolongation [45]. The package insert [46] includes warnings for ECG QT prolongation and ventricular tachycardia (including torsades de pointes) [47]. However, the details of the causal mechanism for these events remain unknown [47]. Currently, guidelines also specify doses to be used with caution in the event of ECG QT prolongation [48].
- Cluster 7: Disorientation, restlessness, and delirium. These adverse events were clusters that were more frequently associated with methadone and tapentadol than with other strong opioids used in palliative care. The hypothesis that delirium, the name of the adverse event, is caused by an imbalance of substances in the brain has been proposed, but no clear mechanism is known [49]. Future research on the relationship between methadone, tapentadol, and delirium is warranted to determine why side effects differ among opioids despite being mediated by opioid receptors, as well as differences in selectivity for opioid receptor subtypes (μ, κ, and δ receptors); we note the findings in previous studies [31,32,33,34,35,36,37,38,39,40,41]. Furthermore, differences in metabolism and excretion mechanisms among drugs [50] and individual genetic factors for opioids [51] may be the causes of differences in adverse drug reaction trends among the patient populations using the various opioids.

These analyses indicate that cluster analysis using a large database can be effectively used to compare adverse effect propensities among opioids. We believe that such comparisons will facilitate effective drug switching to avoid the risk of adverse events. The association of particular adverse event names with particular opioids may lead to a better understanding of the aforementioned differences in characteristics among opioids. Clearer associations may also help prompt the naming of diseases for which associations of particular symptoms with particular opioids would otherwise remain to be confirmed.

### 3.4. Limitations

Several limitations are inherent in this study. Firstly, the constraints are related to the utilized database. This database encapsulates information on adverse effects derived from spontaneous reports, thus restricting the cases to those recognized as adverse effects. In this inquiry, the total number of patients who utilized opioids remained undetermined, impeding an accurate evaluation of the adverse events. To address this, we endeavored to enhance the analytical value by instituting a filter for the number of reports, thereby bypassing simplistic *p*-value and ROR comparisons. Secondly, mild adverse effects may be under-reported, while severe adverse effects are likely reported with greater frequency, illustrating a phenomenon known as reporting bias, a common characteristic of self-reporting databases. Thirdly, it is a known fact that certain FAERS data can be incomplete, potentially containing blank cells indicative of missing values or inaccurately inputted characters or numbers. Fourthly, the co-administration of multiple drugs complicates the identification of the precise cause of adverse events. The ROR values extracted this time are merely referential, with the hope that they will be validated through subsequent appropriate clinical trials.

## 4. Materials and Methods

### 4.1. Data Table Creation

To extract data on adverse reactions associated with opioid drugs, we used 153,673,177 records of reported data from the FAERS database spanning the years 2004–2020 [23,24,25,26,27,28,29,30]. The data tables were categorized based on their characteristics, including a case information table (demographic), a drug information table (drug), an adverse event information table (reaction), and an underlying disease information table (indication). Information across these tables was linked using registration IDs.

**Figure 2:**
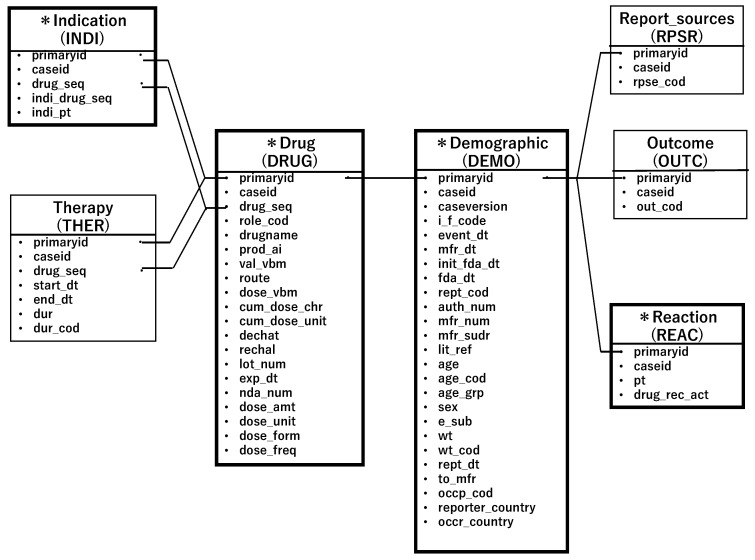
Flowchart for data analysis construction.

The drug table classified the reported drugs into four categories: primary suspect drug, secondary suspect drug, concomitant drugs, and interactions. For this study, we extracted drugs reported as primary or secondary suspect drugs. Each data table was linked by its registration ID to create a data table for analysis for each opioid (see Figure 2).

To selectively extract opioids, we examined target drugs based on their μ-opioid receptor affinity, as reported in previous studies [42,52,53,54,55,56,57,58,59]. We selected 11 opioids, which are μ-opioid receptor agonists used in Japan, including morphine, fentanyl, oxycodone, codeine, dihydrocodeine, hydromorphone, methadone, tapentadol, pethidine, loperamide, and remifentanil. Each opioid extraction case was extracted as the total opioid, including drug names such as hydrate and hydrochloride. For morphine, the drug name excluding apomorphine was extracted as the relevant morphine. For fentanyl, drug names excluding remifentanil were extracted as fentanyl.

To determine the number of adverse event reports for each opioid, we extracted the report counts from the reaction table (see Table 1).

### 4.2. MedDRA

The Medical Dictionary for Regulatory Activities (MedDRA) is an international medical terminology developed by the International Conference on Harmonization of Drug Regulations to facilitate international information exchange and regulatory harmonization. In this study, we used the preferred terms of MedDRA ver22.0 for the disease names of reported adverse events.

From the analysis data table, we created a 2 × 2 contingency table to determine whether all reported adverse events and each suspected opioid were present. This contingency table allowed us to estimate the relationship between the adverse event of interest and the drug of interest (see Table 3). In this study, we employed a combination of the reporting odds ratio (ROR) and *p*-value in Fischer’s exact test as indicators of signal detection. If there are any 0 cells in the contingency table, the ROR calculation cannot be performed, and if the frequency is low, the estimation becomes unstable. To rectify bias, we applied the Haldane–Anscombe 1/2 correction by adding 0.5 to all cells [60]. We then calculated the RORs and Fisher’s exact tests for various adverse events for each opioid.

### 4.3. Hierarchical Cluster Classification

In this study, we used the hierarchical cluster classification method to classify reported adverse events among opioids. This method creates a tree diagram based on the similarity of distinguishing features of objects. We analyzed 21,334 reported adverse events in the reaction table of FAERS and calculated the lnROR for each adverse event of the 11 target opioid drugs. We then extracted 310 adverse event names that were reported >100,000 times.

To identify adverse event names closely related to opioids, we extracted those with a positive mean lnROR value for opioids (Table S1). Subsequently, we performed hierarchical cluster analysis (using Ward’s method) based on the adverse event names and the lnROR of the target opioids (Figure 1).

### 4.4. Statistical Analysis

We performed all statistical analyses using JMP Pro 14.2.0 (SAS Institute Inc., Cary, NC, USA) and set the level of statistical significance at 0.05.

## 5. Conclusions

In summary, the current study analyzed opioid usage in Japan and provided valuable insight into the occurrence of adverse effects related to μ-opioid receptor stimulation by using a novel clustering method for classification. The findings showed obvious differences in the risk of adverse events by opioid strength, and also supported the examination of differences in the incidence of adverse effects by the type of μ-opioid receptor stimulant (including opioids prescribed for pain relief) used. The results of this study can facilitate better decision making with regard to the use of opioids in clinical practices in Japan. Although opioids may be prescribed for palliative and nonpalliative care, the databases used in the current study prevented stratification of the analysis by purpose of use. Future cohort studies and clinical trials should aim to provide more robust evidence in this field by stratifying their analyses into palliative and nonpalliative care. In conclusion, this study provides valuable insight into the relationship between opioid usage and the occurrence of associated adverse events, thus allowing the development of a better understanding of the risks and benefits associated with these drugs in Japan.

## Figures and Tables

**Table 1:** Number of reported adverse events.

Opioid	Number of Reports
Oxycodone	925,184
Morphine	525,135
Fentanyl	449,050
Codeine	315,510
Hydromorphone	259,911
Loperamide	208,812
Methadone	139,804
Tapentadol	61,718
Pethidine	49,948
Dihydrocodeine	29,518
Remifentanil	12,419

Number of reported adverse events for targeted µ-opioid receptor agonists in FAERS.

**Table 2:** Top 47 adverse events.

Adverse Event	Number of Reports	Average lnROR	ROR
PAIN	1,391,676	0.04	1.04
DEATH	1,319,299	0.49	1.62
VOMITING	1,245,241	0.16	1.18
HYPOTENSION	650,888	0.12	1.12
CONSTIPATION	607,289	0.06	1.06
DRUG INTERACTION	570,134	0.29	1.33
SOMNOLENCE	538,091	0.37	1.45
CONFUSIONAL STATE	511,926	0.29	1.34
TOXICITY TO VARIOUS AGENTS	448,332	1.65	5.21
DRUG HYPERSENSITIVITY	440,763	0.53	1.70
OVERDOSE	383,578	1.36	3.88
LOSS OF CONSCIOUSNESS	366,752	0.21	1.23
HYPERHIDROSIS	350,840	0.34	1.40
RESPIRATORY FAILURE	276,741	0.14	1.15
CARDIAC ARREST	263,643	0.61	1.84
TACHYCARDIA	256,046	0.36	1.43
MALIGNANT NEOPLASM PROGRESSION	221,264	0.15	1.17
COMPLETED SUICIDE	212,432	0.50	1.65
AGITATION	205,609	0.39	1.48
BLOOD PRESSURE DECREASED	203,294	0.04	1.04
HALLUCINATION	191,123	0.31	1.36
BRADYCARDIA	183,547	0.09	1.10
LETHARGY	167,887	0.22	1.24
PULMONARY OEDEMA	161,426	0.35	1.41
DRUG ABUSE	159,461	1.73	5.66
INTENTIONAL PRODUCT MISUSE	157,643	0.49	1.63
OXYGEN SATURATION DECREASED	148,131	0.30	1.34
CARDIO-RESPIRATORY ARREST	143,679	0.66	1.93
COMA	140,276	0.60	1.82
DISORIENTATION	134,132	0.35	1.41
FOETAL EXPOSURE DURING PREGNANCY	133,608	0.21	1.23
HYPOXIA	132,851	0.35	1.42
DRUG WITHDRAWAL SYNDROME	124,592	1.01	2.75
DEPRESSED LEVEL OF CONSCIOUSNESS	122,498	0.69	2.00
DYSKINESIA	116,335	0.18	1.19
LIVER FUNCTION TEST ABNORMAL	114,153	0.05	1.05
MENTAL STATUS CHANGES	113,590	0.16	1.17
INTENTIONAL OVERDOSE	112,617	0.29	1.33
ANAPHYLACTIC REACTION	111,092	0.47	1.60
MEDICATION ERROR	108,377	0.48	1.62
DELIRIUM	106,617	0.85	2.35
URINARY RETENTION	106,456	0.09	1.09
EXPOSURE DURING PREGNANCY	105,584	0.24	1.27
RESTLESSNESS	102,856	0.26	1.30
ELECTROCARDIOGRAM QT PROLONGED	100,522	0.24	1.27
HEPATIC FAILURE	100,434	0.08	1.08
WRONG TECHNIQUE IN DRUG USAGE PROCESS	100,201	0.20	1.22

Top 47 adverse events with >150,000 reported cases and associated reporting odds ratio values for target opioids.

**Table 3:** Generation of a 2 × 2 contingency table and calculation of RORs.

	Adverse Event (+)	Adverse Event (−)
Reports with the suspected drugs	a	b
All of reports	c	d

ROR = (a/b)/(c/d) = a × d/c × b.

## Data Availability

Data is contained within the article.

## Supplementary Materials

The following supporting information can be downloaded at: https://www.mdpi.com/article/10.3390/ph16111541/s1, Table S1: Positive mean lnROR value for opioids.
